# Classification of BMI control commands from rat's neural signals using extreme learning machine

**DOI:** 10.1186/1475-925X-8-29

**Published:** 2009-10-28

**Authors:** Youngbum Lee, Hyunjoo Lee, Jinkwon Kim, Hyung-Cheul Shin, Myoungho Lee

**Affiliations:** 1 Department of Electrical & Electronic Engineering, Yonsei University, Seoul, Korea; 2 Department of Physiology, College of Medicine, Hallym University, Chuncheon, Gangwon, South Korea

## Abstract

A recently developed machine learning algorithm referred to as Extreme Learning Machine (ELM) was used to classify machine control commands out of time series of spike trains of ensembles of CA1 hippocampus neurons (n = 34) of a rat, which was performing a target-to-goal task on a two-dimensional space through a brain-machine interface system. Performance of ELM was analyzed in terms of training time and classification accuracy. The results showed that some processes such as class code prefix, redundancy code suffix and smoothing effect of the classifiers' outputs could improve the accuracy of classification of robot control commands for a brain-machine interface system.

## Introduction

A brain-machine interface (BMI) is a communication channel, which transforms a subject's thought processes into command signals to control various devices, for example, a computer application, a wheelchair, a robot arm, or a neural prosthesis. Many studies have been made on the prediction of human voluntary movement intention in real-time based on invasive or noninvasive methods to help severely motor-disabled persons by offering some abilities of motor controls and communications. A noninvasive method records electroencephalographic (EEG) signals and extracts intentional traits or movement-related EEG features, such as the P300 component of an event-related evoked potential [1], EEG mu rhythm conditioning [2-4], or visually evoked potential [5]. Noninvasive methods have low spatial resolution since they take readings from the entire brain rather than a specific part of the brain [6]. On the other hand, an invasive method delivers better signal quality at the expense of its invasive characteristic. Its typical approaches include electrocorticograms [7], single neuron recordings [8], or multi-neuron population recordings [9]. Advanced researches on invasive methods are being actively pursued with the aim of recovering complex and precise movements by decoding motor information in motor related brain areas [10,11]. Naturally, such researches have raised the hopes of paralyzed people. Due to the advances of science and medical technologies, life expectancy has increased. As the person's age increases, the development of multiple chronic conditions increases. The number of motor-disabled and solitary aged people also increases. However, the resources needed to care for the aged is not meeting the demands. A virtual reality linked to a general purpose BMI could be an alternative for the shortcoming resources on the arrival of aging society and the need of assistive technology.

**Figure 1:**
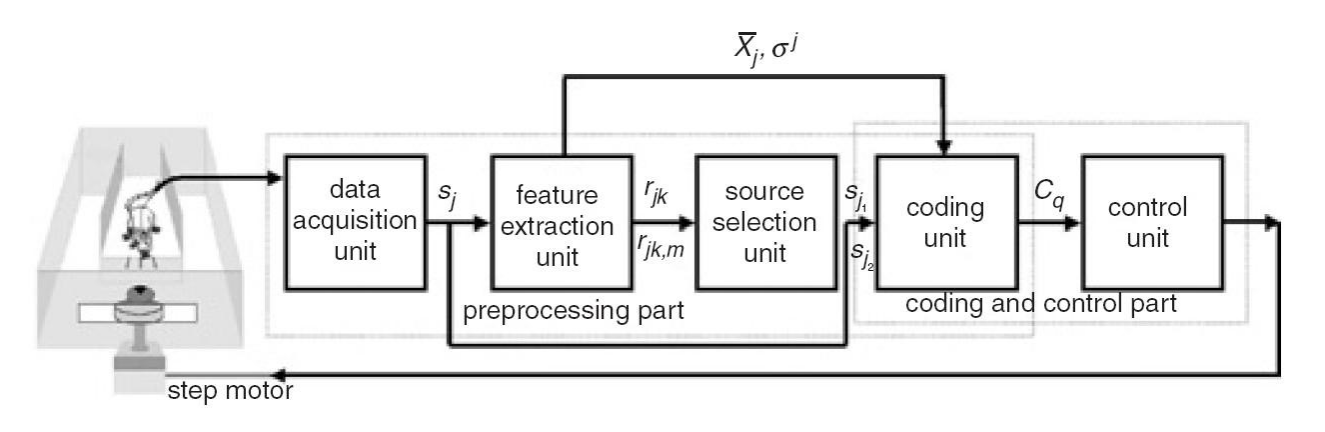
**A block diagram of the BMI system**.

Figure 1 shows a block diagram of the BMI system developed in our previous study [12]. The BMI system was composed of data acquisition, feature extraction, source selection, coding, and control units. In the data acquisition unit, neuronal signals recorded from CA1 region of the rat brain were amplified, filtered, sorted, and transformed into *m *spike trains *s*_*j*_, *j *= 1,2, ⋯, *m *in real-time, where  and  denotes the time of occurrence of the p'th spike emitted by the j'th neuron. Each spike train during a time interval (0, *T*] was transformed into time series data  in the feature extraction unit, where  and *z *= *T*/Δ*t *and Δ*t *= *t*_*i *_- *t*_*i*-1 _is the bin size of the time series data. The neuronal response function *ρ*^*j *^(*t*_*i*_) was evaluated as sums over spikes from j'th neuron for 0 ≤ *t *≤ *i*Δ*t *[13]. The correlation coefficients *r*_*jk *_and the partial correlation coefficients *r*_*jk*,*l *_of the time series data were then calculated using the equations given in reference [14]. The correlation coefficient *r*_*jk *_measures the correlation between the time series data *X*_*j *_and *X*_*k*_. The partial-correlation coefficient *r*_*jk *_measures the net correlation between the time series data *X*_*j *_and *X*_*k *_after excluding the common influence of (i.e., holding constant) the time series data *X*_*l *_[13]. The source selection unit classified the time series data *X*_*j *_into two groups, correlated, and uncorrelated groups, according to the values of the correlation coefficients. Each group was again subdivided into two subgroups based on the values of the partial correlation *s*_*j*1_coefficients of its elements. Two spike-trains  and  were then selected, where the corresponding time series data  and  were belong to the uncorrelated group but not in the same subgroup. In result,  and  were independent each other as well as had large difference in their correlations with other spike trains . The coding unit coded a series of motor functions into the spike train  and  by an coding function  and transformed in real-time the relative difference between the neuronal activities of the spike trains  and  into a command signal corresponding to one of the motor functions. The control unit received the command signal from the coding unit and executed it correspondingly to control a water disk or a robot of the BMI system.

The aim of this study was to see an efficient usability of ensembles, simultaneously recorded many single units for the generation of specified directional commands in a BMI system for a rat to manipulate an external target on a two-dimensional space to achieve rat's volition. For this purpose, ELM was used to classify machine control commands out of time series spike trains of 34 simultaneously recorded CA1 hippocampus neurons.

## Materials and methods

The practical usability and the efficiency of the presented BCI system were tested by experiments of a 'water drinking' task using 11 rats. The subject was to control the degree and the direction of the rotation of the wheel with its neuronal activities of the SI cortex to access water in the WD task. The water was contained in one-quarter of a circular dish positioned on top of the wheel. The experiments were carried out with approval from the Hallym University Animal Care and Use Committee. Adult male or female SPF Sprague-Dawley rats weighing 200-220 g were used. Two multi-wire recording electrodes arrays (eight channels for each array, tungsten microwire, A-M systems, USA, 75 mm diameter, Teflon-coated) were implanted bilaterally into the SI vibrissae area of both right (RH) and left (LH) hemispheres of each rat. Lesions were made to the vibrissae motor cortices in both hemispheres. Infraorbital and facial nerves were bilaterally sectioned to prevent a sensory input from and a motor output to whisker pads. Four weeks after the lesions, the rats were deprived of water for 24 h. Each rat was then placed in front of the wheel to perform the WD task for a trial of an experimental session. Three experimental sessions were carried out over six days for each rat. The rat was deprived of water for 24 h before each session. A session comprised 40 s preprocessing, five 300 s trials, and a 300 s rest period between trials. In the preprocessing, the spike trains from the SI cortex of the rat were assessed by the correlations among them, two spike trains sj1 and sj2 were selected, and then a series of motor functions were encoded into them. The bin size Dt used in the feature extraction unit was 200 ms. A critical value, rc of the correlation coefficient was estimated at the significance level of 0.05 to categorise spike trains to the uncorrelated group, e.g. rc 1/4 0.098 for the sample size n 1/4 400. Seven motor functions were set up for the directions and the degrees of the rotation of the wheel, which were embodied by seven command signals Cq for q 1/4 _3, _2, ..., 3. The absolute value and the polarity of the subscript q of the command signal described the direction and the number of the step of the wheel rotation, respectively. If it was positive, the resulting direction was clockwise (CW), and if negative, in a counter-clockwise (CCW) rotation on the rat side. One-step (C_1) rotation turned the wheel exactly 14.5_, two-step (C_2), 21.5_, and three-step (C_3), 28.5_. In case of zero-step (C0) rotation the wheel was not to turn. During the trials, the relative difference between the neuronal activities of the spike trains sj1 and sj2 were evaluated and categorised into one of the motor functions by the encoding function f (sj1, sj2) and then one of the seven command signals Cq was generated every 200 ms. Then, an Intel i80196 microprocessor in the control unit received the command signal, Cq, and executed it correspondingly. The implanted electrodes to a preamplifier whose outputs were sent to a data acquisition system (Plexon Inc., Dallas, TX, USA) for online multi-channel spike sorting and unit recording.

ELM is a novel learning algorithm for Single hidden Layer Feed-forward Neural networks (SLFNs) which is a kind of artificial neural network. That has the advantage of very fast learning speed and high accuracy [15]. Preceding researches reported ELM can learn thousands of times faster than conventional popular learning algorithms for feed forward neural networks like back propagation neural network (BPNN) without accuracy loss. Because of these advantages, ELM has many possibilities to be adapted to many applications. ELM overcoming defects of BPNN is a novel learning algorithm for SLFN. BPNN is a generally used learning algorithm for artificial neural network among gradient-based algorithm. Gradient-based algorithm adjusts weights between neurons from output layer to input layer. Because of this process, there exists dependency between input weights and output weights. Some researches have shown that SLFNs having including *N *hidden neurons with randomly chosen input weights can learn *N *distinct patterns with randomly small error [6]. ELM is based on this result and has learning process using random chosen input weights and biases of hidden neurons [5]. For approximation of SLFNs, when we have *N *random distinct samples (**x**_*i*_, **t**_*i*_), we can model SLFNs as eq. (1),

(1)

Where **x**_*j *_= [*x*_*j*1_, *x*_*j*2_, ⋯, *x*_*jn*_]^*T*^, **t**_*j *_= [*t*_*j*1_, *t*_*j*2_, ⋯, *t*_*jm*_]^*T *^represents *j *th input vector and output vector, *b*_*i *_is bias of *i *th hidden neuron, **w**_*i *_= [*w*_*i*1_,*w*_*i*2_, ⋯,*w*_*in*_]^*T *^input weight vector connecting *i*th hidden neuron to input layer, **β**_*i *_= [*β*_*i*1_, *β*_*i*2_, ⋯, *β*_*im*_] output weight vector connecting *i*th hidden neuron to output layer, and SLFNs have *N*_*h *_hidden neurons and activation function *g*(·). The eq. (1) is represented by matrix equation as:

(2)

Each component of **H **represent output of hidden layer. When input weights **w**_*i *_and biases *b*_*i *_of hidden neuron are invariable, **H **is determined with input vector **x**_*j*_. In that case, SLFNs are linear system. So, In case of **H **has inverse matrix, we can get **β **through **H**^-1^·**T**. But generally number of samples is greater than number of hidden neurons, **H **is a nonsquare matrix and there may not exist **H**^-1^. The optimal output weights  guarantee minimum difference between **Hβ **and **T **as:

(3)

Using Moor-Penrose generalized inverse **H**^† ^we can get minimum norm least-squares solution of (3).

That case has the optimum value of [5].

The process of ELM for SLFMs learning algorithm is expressed below:

Choose random values for input weights **w**_*i *_and biases *b*_*i *_of hidden neurons.

Calculate hidden layer output matrix **H**.

Obtain the optimal  using  = **H**^†^**T**.

Because learning process of ELM randomly choose the input weights and analytically determine the output weights of SLFNs, there are no iteration processes and that means extremely smaller learning time of ELM than BPNN.

The universal approximation capability of ELM is also critical to show that ELM theoretically can be applied in such applications. ELM has some versions such as I-ELM [16], C-ELM [17] and EI-ELM [18]. I-ELM [16] means incremental ELM. According to conventional neural network theories, single-hidden-layer feed forward networks(SLFNs) with additive or radial basis function (RBF) hidden nodes are universal approximators when all the parameters of the networks are allowed adjustable. However, as observed in most neural network implementations, tuning all the parameters of the networks may cause learning complicated and inefficient, and it may be difficult to train networks with no differential activation functions such as threshold networks. Unlike conventional neural network theories, I-ELM proves in an incremental constructive method that in order to let SLFNs work as universal approximators, one may simply randomly choose hidden nodes and then only need to adjust the output weights linking the hidden layer and the output layer. C-ELM [17] means Complex ELM. C-ELM extends the ELM algorithm from the real domain to the complex domain, and then applies the fully complex extreme learning machine (C-ELM) for nonlinear channel equalization applications. The simulation results show that the ELM equalizer significantly outperforms other neural network equalizers such as the complex minimal resource allocation network (CMRAN), complex radial basis function (CRBF) network and complex back propagation (CBP) equalizers. C-ELM achieves much lower symbol error rate (SER) and has faster learning speed. EI-ELM [18] means enhanced method for I-ELM. An incremental algorithm referred to as incremental extreme learning machine (I-ELM) was proposed by Huang et al. [16]. which randomly generates hidden nodes and then analytically determines the output weights. Huang et al. [16] have proved in theory that although additive or RBF hidden nodes are generated randomly the network constructed by I-ELM can work as a universal approximator. During recent study, it is found that some of the hidden nodes in such networks may play a very minor role in the network output and thus may eventually increase the network complexity. In order to avoid this issue and to obtain a more compact network architecture, this paper proposes an enhanced method for I-ELM (referred to as EI-ELM). At each learning step, several hidden nodes are randomly generated and among them the hidden node leading to the largest residual error decreasing will be added to the existing network and the output weight of the network will be calculated in a same simple way as in the original I-ELM. Generally speaking, the proposed enhanced I-ELM works for the widespread type of piecewise continuous hidden nodes.

## Results

**Figure 2:**
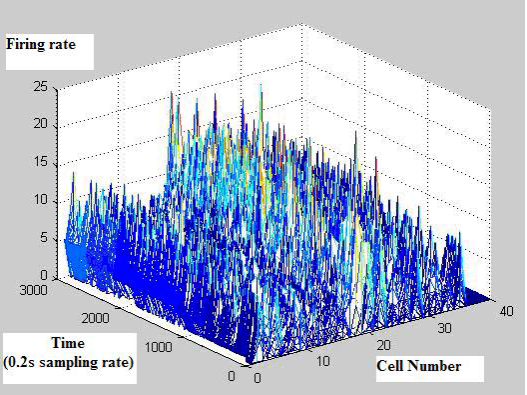
**Rat's neural signal**.

**Figure 3:**
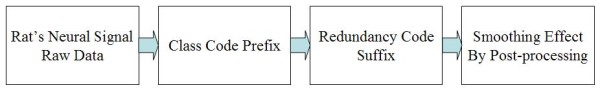
**Performance evaluation process**.

**Figure 4:**
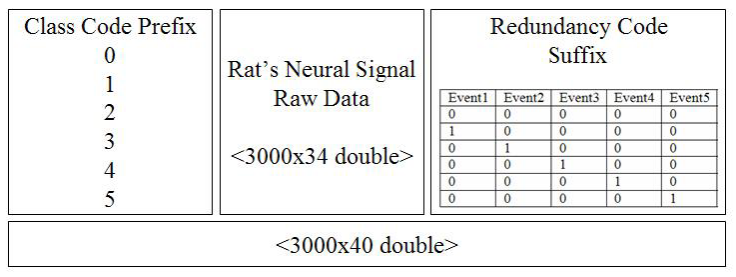
**Modified data format**.

**Figure 5:**
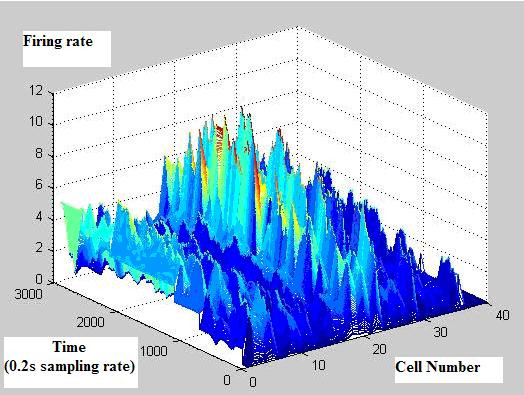
**Smoothing effect**.

**Figure 6:**
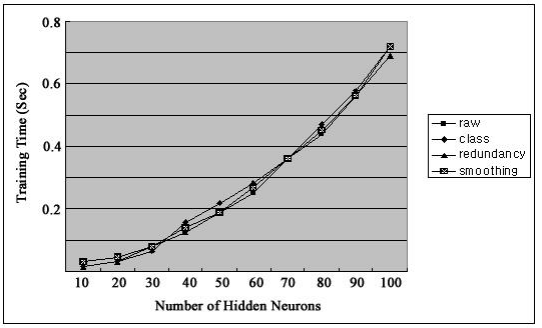
**Training Time**.

**Figure 7:**
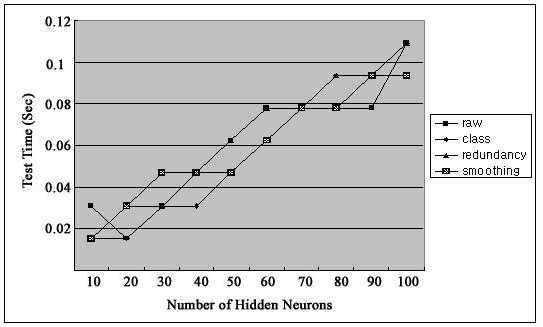
**Testing Time**.

**Figure 8:**
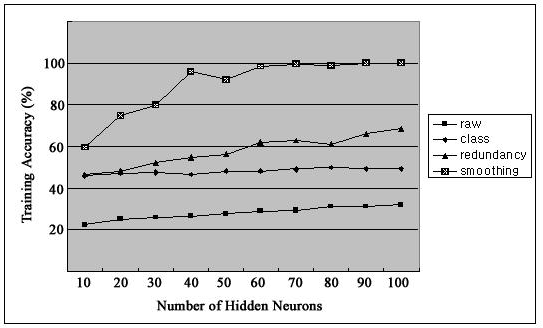
**Training Accuracy**.

**Figure 9:**
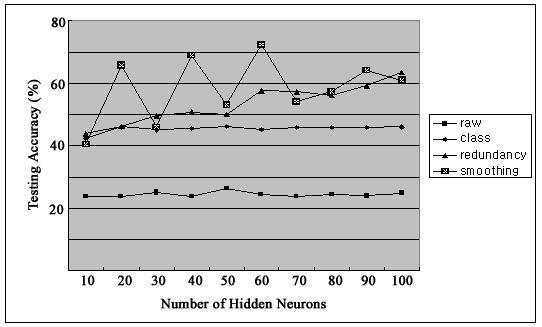
**Testing Accuracy**.

**Figure 10:**
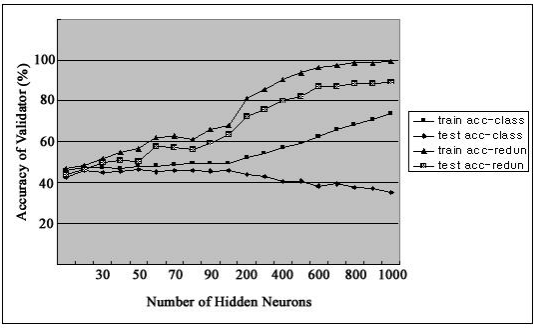
**Class code and redundancy**.

Figure 2 shows firing rates of CA1 single units used in this study. The data means sorted cells' firing rate at 200 ms sampling rate. Spike trains of simultaneously recorded 34 single units for 10 min were used for ELM training (Figure 3) and those for another 10 min were used for testing purpose (Figure 4). When we executed classification for raw data, 3000 samples by 34- sorted cells, using ELM classifier, the accuracy of validation was just below 30% (Figure 5). Therefore, we made several processes for enhancing the classifier performance (Figure 6). First, we allocated class code 0 ~ 5 by 5 event bits such as Event1 ~ Event5 in table 1. Actually, each event bit meant the robot control commands from rat's neural signals such as directions (forward, backward, right, left) and steps. We put the class code column as prefix of the raw data (Figure 7). As the effect of class code prefix, the accuracy level was doubled and became almost 50% as shown in figures 8 and 9. However, when we increased the number of hidden neurons, the training accuracy increased continuously, but the testing accuracy decreased as illustrated in figure 10. Thus, we made some redundancy code as suffix of event bits to raw data. We already allocated class code by event bits, but we left the event bits for enhancing classifier's performance. In this way, the final data format became 3000 samples by 1 class code column, 34 sorted cells and 5 redundancy event bits as depicted in figure 4. As the effect of redundancy code, when the number of hidden neurons is increasing, the accuracy level increased almost linearly as shown in figures 8, 9 and 10. Lastly, we tried to enhance the classifier performance by post-processing such as smoothing the classification algorithm's raw outputs. Figure 5 shows the smoothed data using moving average filter. In figure 8, by smoothing effect, the training accuracy became almost 100%. However, as illustrated in figure 9, testing accuracy appeared to be very unstable and reached only 60% when number of hidden neurons was increased.

**Table 1:** Class code allocation for 5 Events.

**Event1**	**Event2**	**Event3**	**Event4**	**Event5**	**Class**
0	0	0	0	0	0

1	0	0	0	0	1

0	1	0	0	0	2

0	0	1	0	0	3

0	0	0	1	0	4

0	0	0	0	1	5

It seems strange to add class code prefix and redundancy code suffix into the raw data for constructing input vectors of the ELM algorithm or other learning algorithms. But the code prefix or the redundancy code suffix is feature vector for effective pattern classification not the target label or target vector which the algorithm is supposed to learn/predict. ELM algorithm extracts the feature vector from input vectors and in the testing phase, it evaluates the classification performance for output vectors using feature vector.

**Figure 11:**
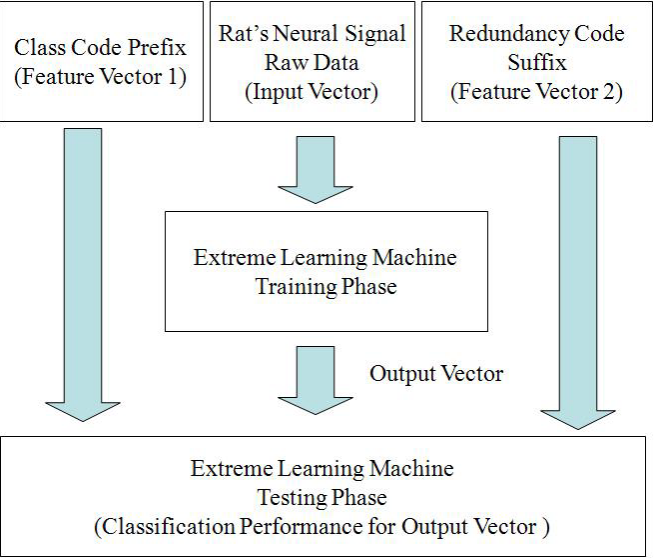
**The classification procedure using Extreme Learning Machine**.

**Figure 12:**
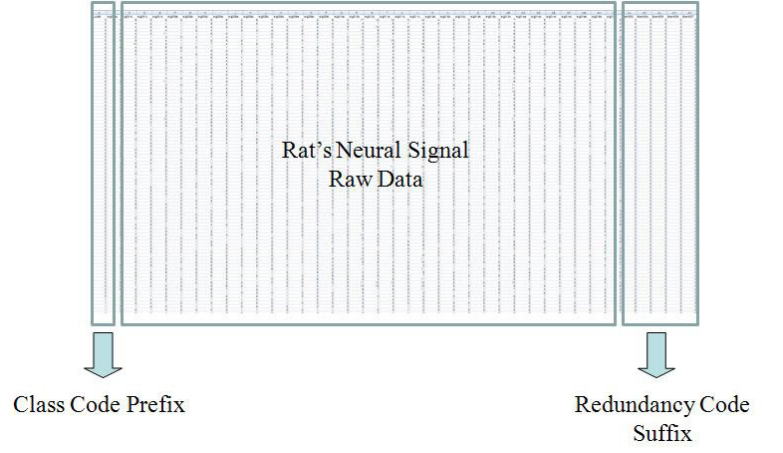
**Real Raw Data**.

In Figure 11, 12, we make some example that shows the classification procedure using Extreme Learning Machine. In Figure 11, the input vector is Rat's Neural Signal Raw Data. ELM uses this input vector in training phase. Moreover, in testing phase, ELM evaluates the classification performance for output vectors using two Feature Vectors (Class Code Prefix, Redundancy Code Suffix). Figure 12 shows real raw data that consists of Class Code Prefix, Rat's Neural Signal Raw Data and Redundancy Code Suffix). Output vector is treated as internal process in ELM. Therefore, we can obtain final classification performance from ELM.

## Discussion

In this study, a recently developed machine learning algorithm [19] referred to as Extreme Learning Machine (ELM) was used to classify machine control commands, such as directions (forward, backward, right, left) and steps, out of time series spike trains of 34 simultaneously recorded CA1 hippocampus neurons. Performance of ELM was analyzed in terms of training time and classification accuracy. The study showed that some processes such as class code prefix, redundancy code suffix and smoothing effect of the classifiers' outputs can obviously improve the classification accuracies of the commands used for the BMI system [20].

In this study, at first, using the ELM classifier, the accuracy of validation was just below 30%. This was quite natural since commands of our BMI were encoded in every 200 ms by two neurons, such that one was for direction and the other for distance. The rest of 32 neurons were not directly used for BMI machine control. The 30% of validation accuracy may suggest that about 1/3 of simultaneous recorded CA1 neurons in the vicinity of the two neurons directly encoding commands were synchronously active in every 200 ms [21].

Our results showed that adding class code column as prefix of the raw data doubled the training accuracy up to 50% with incremental accuracy validation, but reduced validation of testing accuracy as increasing the number of hidden neurons. This class code insertion appeared to increase the tendency of other 32 neurons to behave in synchronous to the two neurons, which were directly responsible for command generation. However, their heterogeneous characteristics shaped by continuous interactions with other modulation inputs [22], i.e., hidden neurons of ELM, in the CA1 circuits might act against the increase of testing accuracy for command generation.

The results of the current study demonstrated that adding redundancy event bits in addition to the class code prefix dramatically increased the classification accuracy especially when increasing the number of hidden neurons. This feature of ELM could be used as a new BMI command generation algorithm to either supplement or replace the current threshold algorithm, where neural firing rates during every 200 ms were classified by manually as one of four activity ranges. This may increase the efficiency of the BMI system, which may reduce the time for rat to utilize the system for its own volition [23].

However, there are many things to be done in future studies. First, we need to obtain testing accuracy for each event such as directions (forward, backward, right, left) and steps. Second, it is necessary to make a comparison table for each event that shows the correlation between actual activities and estimated activities. Third, additional performance evaluation parameters such as the sensitivity and specificity should be calculated. Lastly, it is necessary to compare the results of ELM methods to other classifiers such as BPNN [24], support vector machine [25] and evolutionary ELM [26].

## Competing interests

The authors declare that they have no competing interests.

## Authors' contributions

YL carried out the rat's neural data analysis, participated in the classification BMI control commands using ELM and drafted the manuscript. HL carried out the BMI experiments using rat. JK participated in the classification BMI control commands using ELM. HS participated in the design of the study and performed the statistical analysis. ML conceived of the study, and participated in its design and coordination. All authors read and approved the final manuscript.
